# Direct exposure of the head to solar heat radiation impairs motor-cognitive performance

**DOI:** 10.1038/s41598-020-64768-w

**Published:** 2020-05-08

**Authors:** Jacob F. Piil, Lasse Christiansen, Nathan B. Morris, C. Jacob Mikkelsen, Leonidas G. Ioannou, Andreas D. Flouris, Jesper Lundbye-Jensen, Lars Nybo

**Affiliations:** 1 Department of Nutrition, Exercise and Sports, Section for integrative physiology, University of Copenhagen, 2200 Copenhagen N, Denmark; 2 Danish Research Centre for Magnetic Resonance, Center for Functional and Diagnostic Imaging and Research, Copenhagen University Hospital Hvidovre, 2650 Hvidovre, Denmark; 3 FAME Laboratory, School of Exercise Science, University of Thessaly, Thessaly, Greece

**Keywords:** Cognitive neuroscience, Neurophysiology

## Abstract

Health and performance impairments provoked by thermal stress are societal challenges geographically spreading and intensifying with global warming. Yet, science may be underestimating the true impact, since no study has evaluated effects of sunlight exposure on human brain temperature and function. Accordingly, performance in cognitively dominated and combined motor-cognitive tasks and markers of rising brainstem temperature were evaluated during exposure to simulated sunlight (equal to ~1000 watt/m^2^). Acute exposure did not affect any performance measures, whereas prolonged exposure of the head and neck provoked an elevation of the core temperature by 1 °C and significant impairments of cognitively dominated and motor task performances. Importantly, impairments emerged at considerably lower hyperthermia levels compared to previous experiments and to the trials in the presents study without radiant heating of the head. These findings highlight the importance of including the effect of sunlight radiative heating of the head and neck in future scientific evaluations of environmental heat stress impacts and specific protection of the head to minimize detrimental effects.

## Introduction

Approximately half of the global population live in regions where environmental heat stress is an issue that annually affects the ability to live healthy and productive lives^1,2^. Negative effects range from thermal discomfort and impaired physical work capacity for healthy adults^3^ to increased morbidity for workers regularly experiencing hyperthermia^1^ and higher mortality for vulnerable citizens during heat-waves^4,5^. While these issues are well-documented and exposure likely to be worsened by global warming, the effect of environmental heat stress on performance in cognitively dominated tasks is less clear. Intriguingly, in laboratory experiments, impairments in cognitively dominated task performance emerge only with profound hyperthermia^6–10^, whereas productivity losses in occupational settings are reported at considerably lower temperatures^1,11^. The ability to maintain concentration and avoid attenuation of motor-cognitive performance is certainly of relevance for work and traffic safety as well as for minimizing the risks of making mistakes during other daily tasks. However, the lack of ecological test validity^1,12–14^ as well as the low sensitivity of the stereotypical test protocols used in previous clinical or mechanistic studies in controlled indoor settings may have underestimated the impact of environmental heat stress in real-life scenarios^6–10^. In particular, although laboratory-controlled environmental heat-stress studies commonly investigate or control for temperature, humidity and wind speed, solar radiation is often neglected, despite contributing a substantial heat load in outdoor settings, especially in the upright position with the human head directly exposed to solar radiation^15^.

Human brain function is highly dependent on a stable supply of blood glucose and oxygen, but the brain is also highly vulnerable to alterations in the ability to maintain cerebral heat balance^16,17^. This vulnerability stems from the brain’s increased metabolic heat production, being highly insulated due to its enclosure within the cranium, and relying primarily on heat removal via the cerebral perfusion for heat loss, thereby causing average brain temperature to be highly dependent on arterial blood temperature^16^. During resting conditions, cerebral thermal homeostasis is maintained by removing ~1 kJ/min (the cerebral metabolic heat production) via the cerebral blood flow as the ~700 ml/min of blood entering the brain via the four feeding arteries leaves the brain with a 0.3 °C higher temperature^17^. Consequently, cerebral heat loss is compromised in hyperthermic humans, as blood flow to the brain decreases while the temperature of incoming blood simultaneously increases^16^. Indeed, the body core-brain temperature relationship is remarkably difficult to alter and neither substantial extra-cranial cooling with ice packages or face fanning^18^, nor lowering the intranasal cavities temperature^19^ affects this relationship, and only some light has been shed regarding the involved central fatiguing mechanisms^3,17^. Notwithstanding, direct exposure of the head to solar radiation might increase cerebral temperature beyond the core temperature, as broad-spectrum light may penetrate some millimeters into skin, and heat the underlying tissue^20^. However, heat flux through the cranial bone is limited^21^ and it remains speculative if cerebral functions and brain temperatures are affected.

Therefore, the present study investigated how direct exposure of the head to simulated solar radiation, compared to solar radiation applied to the lower-body, affected motor-cognitive task performance and markers of deep brainstem temperature changes. To asses motor-cognitive task performance, we applied a test battery (see Supplementary Material for detailed description of the protocol) consisting of a cognitively dominated task (math calculation), simple and complex motor tasks (pinch-force control), and a combined motor-cognitive task (math calculation with force control). To separate the effects of specifically heating the head from general body core hyperthermia, we used the interval between early peaks of the brainstem auditory evoked potentials (BAEPs), as this measurement is a sensitive marker for deep brain temperature changes^18,22^. We hypothesized that direct solar exposure to the head would impair performance in the combined motor-cognitive test battery, either by heating the entire brain or via effects confined to cortical areas.

## Results

### Impact of acute and prolonged exposure to simulated sunlight on motor-cognitive performance

Impaired scores for the simple, complex motor and combined motor-cognitive tasks signified that prolonged exposure of the head to simulated sunlight had considerable consequences for a performance in a range of motor-cognitive tasks. In contrast, neither acute exposure nor matched elevation of the deep core temperature, provoked by lower-body heating, was associated with such deteriorations of motor-cognitive performance.

**Figure 1:**
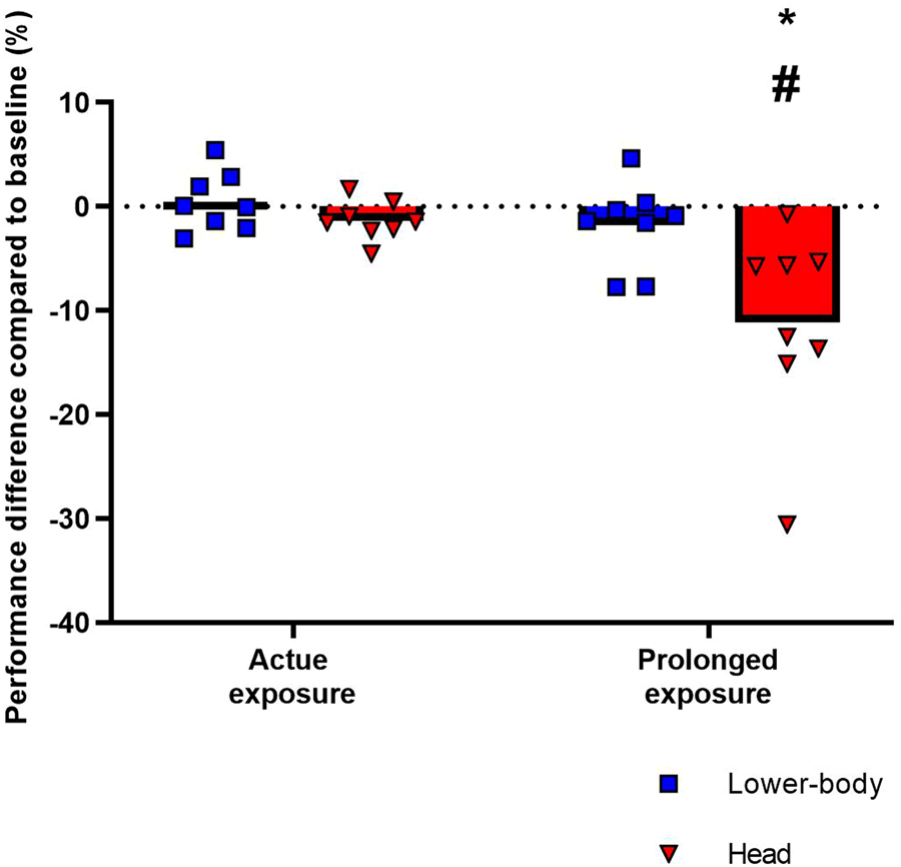
Complex motor task performance. Tracking performance expressed as percentage changes compared to baseline without radiation (delta change from the horizontal black line). Blue bars represent average values from trials with radiation applied to the lower-body and red bars represent trials with radiation applied directly to the head (see Supplementary Material for details). Error bars represent standard deviations (SD). * denotes a significant difference between prolonged and acute exposure, # denotes a significant difference between radiation applied to the head and lower-body (both P < 0.05).

As illustrated in Fig. 1, complex motor performance (the skilled visuomotor tracking task) was not affected by acute exposure, but a time x group interaction (P = 0.021) was observed. Specifically, prolonged exposure of the head to simulated sunlight impaired visuomotor tracking performance by 11.2 ± 9.3% (P = 0.002 compared to baseline) and by 9.4 ± 10.1% when performance is compared to the parallel trial with prolonged radiation exposure of the lower-body (P = 0.008).

No time x trial interaction was observed for the combined motor-cognitive (P = 0.450) or for the simple motor task (P = 0.066). However, main effects of time (P = 0.002) and group (P = 0.016), were observed for the combined motor-cognitive task and a main effect of time (P = 0.006) was observed for the simple motor task. The average across trial decrease in performance after prolonged simulated solar radiation was 7.0 ± 6.6% (combined motor-cognitive task) and 2.1 ± 2.4% (simple motor task). No changes in performance (P > 0.05 at all time-points in both trials) were observed for the task relying on mathematical calculation and typing of the computed sum (the task considered as cognitively dominated).

### Association between core temperature with latency changes in brain stem auditory-evoked potentials

**Figure 2:**
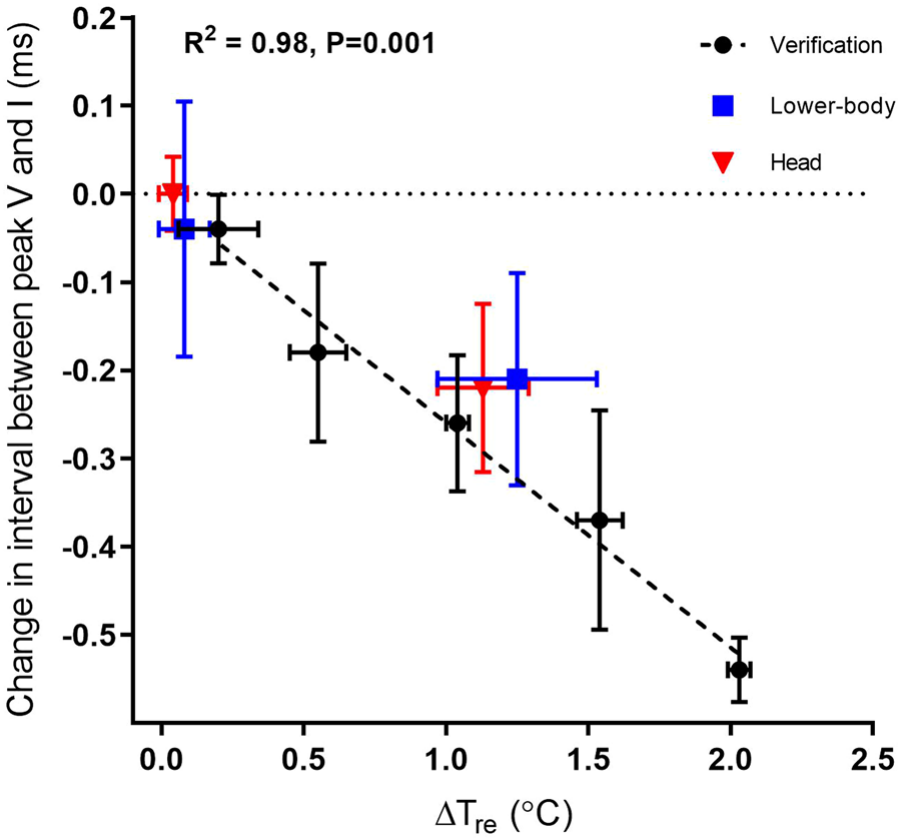
Changes in interval between peak V and I in brainstem auditory-evoked EEG potentials plotted against changes in rectal core temperature. Correlation between changes in interpeak interval (PV-PI) and gradual increase in rectal core temperature from baseline/no radiation (black horizontal dotted line, expressed as 0.0) to the maximal tolerable rectal core temperature/prolonged simulated sunlight exposure. Black dots with error bars (verification session, n = 5, R^2^ = 0.98 expressed as black dashed line, P = 0.001), blue squares with error bars (lower-body radiation session, n = 8) and red triangles with error bars (head radiation session, n = 8). Values are mean and error bars (horizontal and vertical) represent SEM.

**Figure 3:**
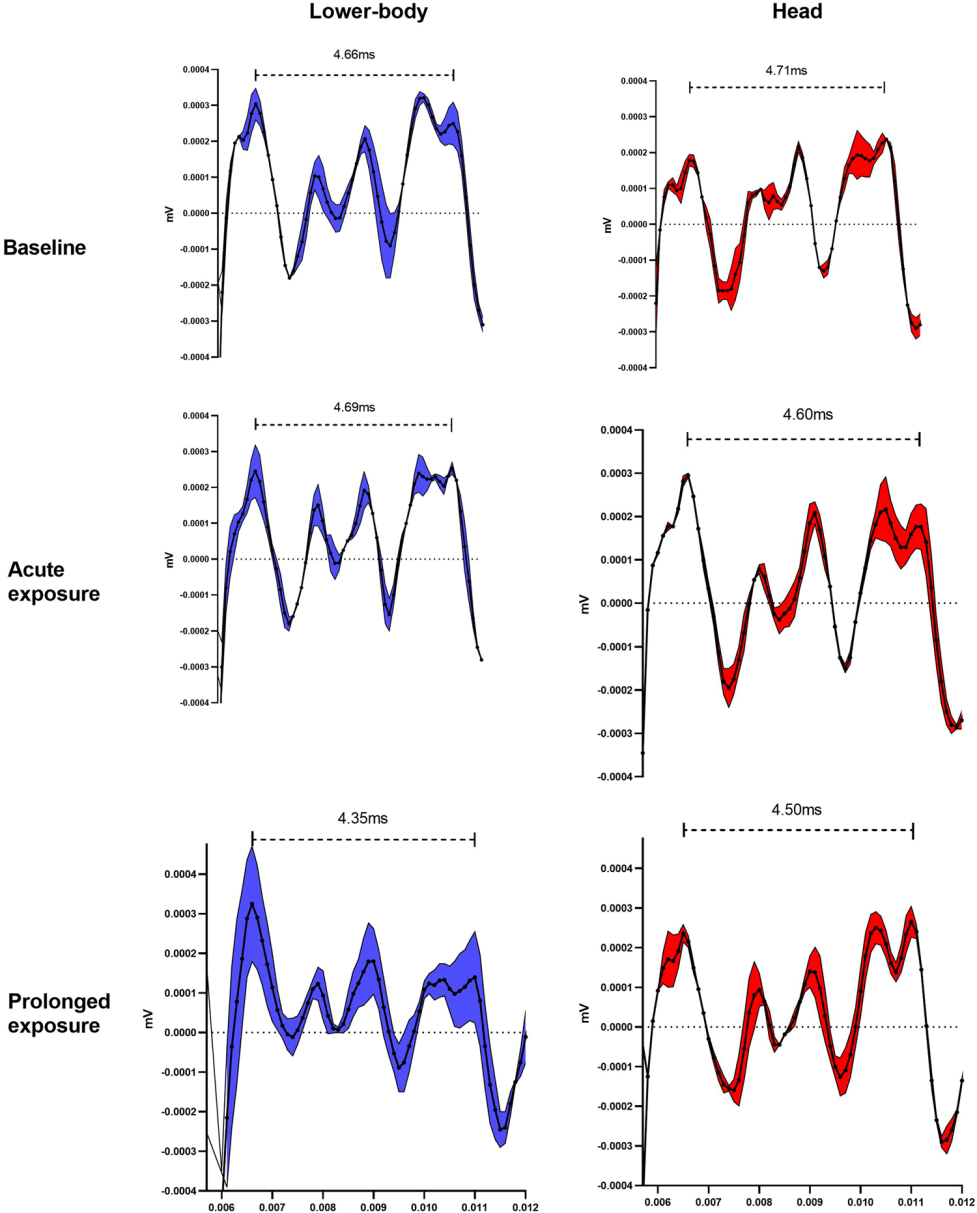
Visual representation of brain stem auditory-evoked potentials from one representative participant. Visual illustration of the brainstem audiotory-evoked potentials across time-points (baseline, acute and prolonged exposure) and sessions (lower-body and head) and the interval time changes (between peak V and I) following exposure time-points. Black dotted solid line represent the average BAEP trace for 1500 stimuli assessed in duplicate before exposure to radiation (baseline - top panel), after 15 min (denoted acute exposure - middle panel) and after prolonged exposure (bottom panel – subjects exposed for ~ 2 hours until the core temperature was elevated by 1 degree above baseline) in the “lower-body radiation trial” (blue) and “head radiation trial” (red). Blue and red area around the black dotted line, represent the SEM of the two obtained BAEP measures at each time-point. Horizontal black dotted line represent 0 mV and black dashed line with ends (above each drawings) shows the absolute interval in milliseconds (ms) between peak I and V for each time-point. X-axis in seconds (s) and y-axis in millivolts (mV).

A verification session was performed to establish the relationship between elevations in core temperature (in steps of 0.5 °C, induced with non-radiative warming), and shortening of the interval between peak V and I (interpeak interval, IPI) of the BAEP. In agreement with previous observations^18,22^, this trial identified an inverse linear relationship between rectal core temperature and shortening of the IPI of the BAEP (see Fig. 2; black dots with dashed line). On average, the interval between peak V and peak I (see Fig. 3 for visual representation of a the BEAP of a single representative subject and Supplementary Material for details) decreased by 0.26 ms per degree increase in core temperature. A similar shortening of the IPI of the BAEPs, was observed both when prolonged (~2 hours until the core temperature was elevated by 1 degree above baseline) simulated solar radiation was applied directly to the head (Fig. 2; red triangles) or to lower-body (Fig. 2; blue squares), and provoked a ~1 degree elevation in the participants’ rectal core temperature. In contrast, no change in IPI of the BAEPs or elevations of rectal core temperature occurred with acute (15 min) exposure (see Figs. 2 and 3).

### Physiological and psychological heat strain

Head skin temperature (HST) and lower-body skin temperature (LBST) were similar between sessions at baseline, but displayed significant changed over time and with a clear time x trial interaction (P < 0.0001). Specifically, direct exposure of the head induced an immediate and prolonged elevation of HST (P < 0.001); while LBST was elevated only in the trial with lower-body exposure (P < 0.001), with no difference between acute and prolonged exposure (see Table 1). A main effect of time (P < 0.0001) was observed for thermal discomfort (TDC) and thermal sensation (TS), with an immediate increase in both trials following acute exposure and was further aggravated following prolonged exposure (with no differences between trials see Table 1). Likewise, a main effect of time (P < 0.0001) was observed for heart rate (HR) and core temperature, which changed similarly across the two sessions and maintained hydration was secured in all experimental conditions by matching water intake to sweat losses, and was verified by maintained body weight and unaltered USG (P > 0.05, urine specific gravity as measure of hydration; see Table 1).

**Table 1:** Measures of physiological and psychological strain at no radiation, acute exposure and prolonged exposure during radiation applied to the lower-body and head.

	Radiation applied to the lower-body			Radiation applied to the head		
	no radiation	acute exposure	prolonged exposure	no radiation	acute exposure	prolonged exposure
Tre (°C)	37.2 ± 0.2	37.3 ± 0.2	38.5 ± 0.4* #	37.3 ± 0.2	37.3 ± 0.2	38.4 ± 0.2* #
HR (bpm)	71 ± 11	77 ± 15	115 ± 17* #	76 ± 10	77 ± 11	112 ± 23* #
Body Mass (kg)	85.0 ± 13.7	—	85.0 ± 13.8	85.0 ± 14.4	—	84.6 ± 14.4* ^
USG	1.014 ± 0.009	—	1.014 ± 0.010	1.015 ± 0.005	—	1.017 ± 0.007
MST (°C)	35.9 ± 0.2	36.9 ± 0.5	38.2 ± 0.6*	35.5 ± 0.5	36.1 ± 0.1	37.8 ± 0.3*
HST (°C)	36.4 ± 0.7	36.6 ± 0.5	37.1 ± 0.7	36.3 ± 0.7	40.7 ± 1.5* ^	41.7 ± 0.8* ^
LBST (°C)	34.1 ± 1.1	42.2 ± 1.1* ^	42.6 ± 1.2* ^	35.0 ± 0.8	35.4 ± 1.0	36.6 ± 1.0
TDC (%)	28.8 ± 15.3	48.3 ± 12.8*	76.9 ± 15.9* #	33.1 ± 16.1	55.3 ± 18.6*	81.2 ± 15.8* #
TS (%)	67.9 ± 6.3	77.3 ± 6.3*	90.9 ± 4.9* #	73.4 ± 81.2	81.2 ± 9.0*	94.9 ± 4.9* #

Mean values across sessions and time-points: T_re_ (rectal temperature in degree Celsius), HR (heart rate in beats per minute), body mass (BM, kilograms), USG (urine specific gravity), MST (mean skin temperature in degree Celsius), HST (head and neck skin temperature measured at the occipital bone), LBST (lower-body skin temperature meased at lumbar spine), TDC (thermal discomfort in % of high [i.e. very uncomfortable 100% to very comfortable 0%]) and TS (temperature sensation rating in % of high [i.e. very hot 100% to very cold 0%]). Values are mean ± SD. * significant different from no radiation, # significant different from acute exposure and ^ significant different from the corresponding session (lower-body or head) session (all P < 0.05).

## Discussion

The present study provides proof-of-principle that prolonged radiant heat exposure of the head impairs motor-cognitive performance, with small but significant impairments in the simple motor task performance and large performance decrements in the complex motor and combined motor-cognitive task. In contrast, such impairments were not provoked when radiative exposure of the lower-body elicited a similar elevation in the deep core temperature and a comparable change in the marker for rising brainstem temperature; thereby indicating that direct exposure of the head and neck to radiant heat mainly affects the superficial parts of the brain. Short-term exposure of the head and neck immediately elevated extra-cranial (skin) temperature but did not influence the measures of deep core temperature or markers of increased brainstem temperature, and all measures of motor-cognitive performance remained unaffected with acute exposure. In agreement with the maintained performance in the lower-body exposure trial, previous studies have demonstrated that moderate hyperthermia^6,9^ with or without accompanying dehydration^23^ has minimal impact on motor-cognitive task performance. In contrast, the substantial performance decrements in the head and neck exposure trial resemble the effects observed during severe hyperthermia^6–9,24^, consequently underlining the importance of incorporating solar radiation into analyses of environmental heat stress impact on human performance.

In terms of ecological outdoor settings, the present study is highly relevant for the billions of people living in areas with permanent or seasonal heat stress, including high levels of solar radiation, which are not currently accounted for in weather analyses based on climate service data (e.g. meteorological forecasts or projected effects of climate change on [shaded] air temperature^25,26^). Therefore, the negative effects on motor-cognitive performance observed in our study indicate that the true impact of climate change may be markedly underestimated if only air temperature is accounted for^27^, or if analyses are based on findings from laboratory studies with low or no radiant heat stress. In addition, impairments in real-life settings following direct sunlight exposure could relate to disturbing of the visual input i.e. sunlight blinding workers. However, visual interference was carefully prevented by sheltering the participants’ eyes from the lights (*see experimental set-up in* Supplementary Material) and the unchanged performance with acute exposure further emphasizes that visual inputs was not affected by illumination or blinding effects. In addition, the marked elevation of extra-cranial temperatures without body core hyperthermia (following acute exposure of the head; see Table 1), did not disturb the overall thermal homeostasis or motor-cognitive performance, whereas deteriorations appeared when elevated extra-cranial temperatures were combined with moderate body core hyperthermia.

The robust correlation between shorter IPIs of the BAEP and elevations in the body core temperature (illustrated on Fig. 2 for the non-radiative verification trial, lower-body, and head radiation exposure experiments), support the notion that the average brain stem temperature is highly dictated by the temperature of the inflowing arterial blood^16^. Furthermore, no performance effects were observed with acute exposure of the head or prolonged lower-body exposure, but emerged when radiation towards the head was combined with an elevated core temperature. It may therefore be hypothesized, that the observed decrements in performance following sunlight exposure affected superficial brain areas and the potential for heat removal via the circulation was compromised by the increased core temperature. Solar radiation, as well as the simulated sunlight applied in the present study, contains a spectrum of light with the main intensity of wave-length from 300–800 nm (see Supplementary Materials for more details). It is likely that broad-spectrum light will penetrate some millimeters into skin^20^ and influence the subdermal tissue temperature, although it appears that approximately 90% of incoming light radiation is scattered and/or absorbed by the skin regardless of skin color^28^. Heat flux through the cranium is much lower, but considering that the cortical bone has a thermal conductivity of ~0.68 W/m·°C^21^, it is likely that some heat penetrated the skull and influenced thermal balance in cortical areas when prolonged radiation provoked a ~5 °C elevation of the skin located proximate to these areas^22,29–31^.

Epidemiological studies have identified that the risk for work-related injuries and accidents increase during periods with elevated environmental heat stress^32,33^ and these incidents may be aggravated if exposure to solar radiation is superimposed^34^. The causal link for these incidents cannot be derived from such observational studies, since superimposed solar radiation may have a direct effect and influence the overall heat-load (integrated effect of air temperature, humidity, windspeed and radiation)^35,36^, which will consequently exaggerate the core temperature response for a given workload, provoke fatigue, and worsen thermal discomfort^36,37^. These high core temperature-derived consequences may be of particular relevance for sporting activities and very physically demanding occupations. However, the majority of outdoor workers only experience moderate elevations in the deep body temperature during a working day^1,11,32,38^ and many job tasks do not involve strenuous physical work but depend on fine motor coordination, cognitively-dominated performance or combined motor-cognitive tasks; i.e. tasks that to some extend resemble the tests completed by the participants in the present study. The eventual negative effect of heat stress on cognitive performance may be highly scenario-specific, and may be mitigated by reducing radiative exposure of the head (with hats or shading) or aggravated if the ability to dissipate heat from the head is restricted, e.g. when safety helmets or other headgear is required. In the translation to ecological settings, it should also be considered that the participants in this study were volunteers, capable and willing to complete the repeated prolonged heat sessions (required to allow time for performance testing and completions of BAEP traces). These sessions were, as reported, associated with profound thermal stress and discomfort, that heat-insensitive individuals would most likely not volunteer for, and less fit subjects might not be able to tolerate^39^. Larger effects, or effects emerging at lower heat stress levels, may therefore be expected in a mixed population, but the present findings are considered very relevant for the billions of people seasonally exposed to environmental heat stress^1^ and for analyzing effects on human productivity and work safety across a range of occupational settings^34^ as well as the ability to perform daily activities^40^.

In the present “proof-of-principle” study, effects of head versus lower-body exposure were evaluated only in healthy young males, as we, for ethical and standardization reasons, the elderly^41,42^, heat-vulnerable populations^43^ and females^44–46^, were excluded from this study. Both, the defined inclusion criteria and additional selection bias (related to the unlikelihood that subjects with low heat tolerance would volunteer for such studies), may imply that larger effects could be observed in a mixed population. This is a general issue in studies with volunteering participants and there is a general underrepresentation of investigations involving females in heat-studies. However, impaired motor-cognitive performance has been reported in both those including both women and men^7,47–52^.

In conclusion, the present study demonstrates that prolonged exposure of the head and neck to simulated sunlight impaired performance in a range of tasks relying on combined motor-cognitive functions and with deteriorations emerging at noticeably lower levels of hyperthermia than previously reported for experiments not accounting for the radiative heat exposure effect. Considering that, except for spinal reflexes or philosophical tasks, human performance is indeed dependent on combined motor-cognitive functions, and considering that solar exposure is a significant factor in many ecological outdoor settings, these results indicate it is important to include the detrimental effects of solar radiation in future analyses of current weather or climate change effects on workers in occupations exposed to high levels of solar radiation.

## Methods

### Participants

Eight recreationally healthy active males (age: 34 ± 7 years, body mass: 85.0 ± 13.7 kg, height: 181.5 ± 7.3 cm) participated in the study. All participants were instructed to be adequately hydrated (see guidance and verification procedures with USG measurements and body weighing below) abstain from alcohol and strenuous exercise 24 h before any scheduled experiments. All participants received written and oral information of experimental procedures about any risks and discomfort associated with the experiment, before they provided written consent to participate in this study, approved by the National Committee on Health Research Ethics *(protocol number: 55907_v3_02012017)* and therefore in accordance with the Declaration of Helsinki.

### Experimental design

*Verification of changes in brain stem evoked potentials latency (BAEP) with gradual increased core temperature:* Before commencing the familiarization- and test sessions, five participant (later enrolled in the primary study) engaged in a varification protocol, to verify decrease in interpeak intervals (PV-PI) in BAEP with a gradual increase in rectal core temperature, similar to what has been observed previously^18^. All participants arrived to the laboratory approximately 20 min before the initiation of the verification protocol. Participant sat down on a backless chair in a neutral environment and for BAEP measurements, silver disk electrodes were placed on the forehead (ground), on the vertex (CZ) and on the right earlobe (described below). Participants then entered the environmental chamber, sat down on a backless chair and baseline BAEPs were recorded. After, the initial recordings, participants shifted position to an ergometer bicycle (Monak E839, Sweden), and cycled until core temperature was elevated 0.5 °C above baseline, then returned to the backless chair and BAEP was recorded again. This cycle continued in intervals of 0.5 °C until participants reached ~2 °C above baseline or until reaching their tolerable limit.

#### Overall design

Following a familiarization session to the behavioral motor-cognitive test protocol, brain stem evoked potentials (BAEP), the radiant heat of the lamps and the room temperature (described below), all participants completed two sessions on two different occasions, separated by a minimum of seven days in an environmental chamber at the University of Copenhagen, maintained at 40.8 ± 0.6 °C and 17.6 ± 6.5% relative humidity. Motor-cognitive testing (described below) was performed three times during each session (no radiation, acute exposure [after 15 min of simulated sunlight exposure] and prolonged exposure [~2 hours until the core temperature was elevated by 1 degree above baseline]). During both sessions, lamps (described below) were pointed towards the lower-body and head, and all testing and training sessions (familiarization and experimental sessions) were completed within two month for each participant. Participants were unaware of the researchers’ hypotheses and naive to the purpose of the study, but for obvious reasons, the participants were not blinded to the experimental conditions (e.g. lower-body vs. head radiation).

#### Familiarization session

Before initiating the experimental sessions, each participant was familiarized with all experimental procedures (e.g. cognitive testing, EEG measurements, radiant heat exposure from the lamps etc.), this to avoid significant learning effects in the motor-cognitive protocol during the actual testing sessions, which potentially could obscure the results of the test. All familiarization was conducted in the climate chamber at the same room temperature (40 °C) that the participants would encounter during the experimental sessions, which due to the large heat strain, both physically and perceptually, could potentially cause a selection bias i.e. favoring heat resilient participant over non-heat resilient. Furthermore, the cognitive familiarization protocol consisted of 1 day with 2 × 30 min. of the entire motor-cognitive protocol (e.g. 1 h of familiarization training on the motor-cognitive test battery); every 30 min a participant had a short break of five minutes.

#### Test sessions

Similar to our previous work^6,23,53^, all participants arrived at the laboratory 30–45 min before the start of the experiment at both sessions. Participants then emptied their bladders into a sealed urine container, and individual urine samples were subsequently analyzed for urine specific gravity (USG) using a refractometer. Upon entering the chamber, all participants completed questionnaires on a visual analog scale (VAS), reporting thermal discomfort rating (TDC) and temperature sensation rating (TS) and were weighed (without clothes). The participant then sat down on a backless stool and a measure of resting heart rate (HR) was obtained prior to engaging in the protocol. All participants then relaxed with closed eyes and 1500 BAEPs were recorded (see description below, BAEP recordings). Following an additional short familiarization period (5 min) in order to avoid warm-up decrements in motor-cognitive performance and then a baseline (no radiation) of the motor-cognitive test battery lasting 15 min. Further, during all testing participants were encouraged to ingest adlibitum 37 °C water in order to prevent dehydration without directly influencing core temperature. Following the completion of the no radiation motor-cognitive test (baseline), the heat radiation lamps were turned on and after 5 min participants reported TDC and TS on a visual analog scale and BAEPs were again recorded and participants completed once again the motor-cognitive test battery. Thereafter, participants stayed seated until core temperature was increased 1 °C above resting levels and all procedures were repeated.

During all motor-cognitive tests (specified below: see section *Motor and cognitive performance testing*), participants were seated on an backless chair in front of a 24-inch computer monitor (Samsung; South Korea), wear custom made lensless protective glasses with the ability to screen out the light from entering and disturbing participants vision. The participant used their preferred hand for all of the cognitive tests. The motor tasks involved recording of pinch force via a load cell (Dacell, model UU3-K5, 5 Kgf), connected to an amplifier (Dacell, DN-AM-310). The signal was subsequently digitalized and sampled at 500 Hz, with a data acquisition board, NI-USB-6008 (National instruments Inc., USA). A customized script built on PYTHON (Python software foundation, USA) was used for running the protocol.

### Measurements

#### Motor-cognitive performance testing

The motor-cognitive test protocol utilized in the present study, was heat sensitive and previously validated^6^. The protocol consisted of four different tasks: 1) Simple motor task: a single or 2 digit number (ranging from 1–99) was displayed in the middle of the monitor corresponding to the target force that the participant was required to pinch/adjust force to, with the aid of visual feedback (green line cursor moving across the screen at a constant speed as well as a y-axis ranging the whole spectrum) and the target number changed from one sequence to the next). 2) Simple cognitive task: on the monitor four numbers were displayed and the participants calculated and typed (using the numb pad) the sum of the four numbers). 3) Combined motor-cognitive: similar to the simple cognitive task, however, participants provided the result via motor response/pinched force. For both math tasks, four numbers (from 1 to 25) were displayed in each corner of the monitor. The participants were then required to calculate the sum of the four numbers (math addition) and either type the result (representing a simple cognitively dominated task) or pinch/adjust force via the strain gauge transducer, using thumb and index finger (representing a combined motor-cognitive task). 4) complex motor task with visuomotor tracking (VMT): The participants were instructed to apply pinch force to a strain gauge transducer (with the thumb and index finger) thereby adjusting the vertical position of a cursor moving with constant speed across the monitor to as accurately as possible hit and stay within five target boxes. The task was adapted from Christiansen *et al*.^54^. Each sequence of the test protocol started with the complex motor task, followed by a simple cognitive or combined motor-cognitive task, and finished with a simple motor task. The entire test consisted of 60 “sequences” i.e. 20 series of 3 task sequences (separated by a 3 s break between each sequence). Each of the above-mentioned tasks lasted for 12 s and following each task, visual feedback (1 s) of the performance was provided with the score appearing in the bottom right corner of the screen and 2 s of transition time to the next task, and the total sequence duration equaled 15 min.

#### Brain stem auditory evoked potential (BAEP) recordings

**Figure 4:**
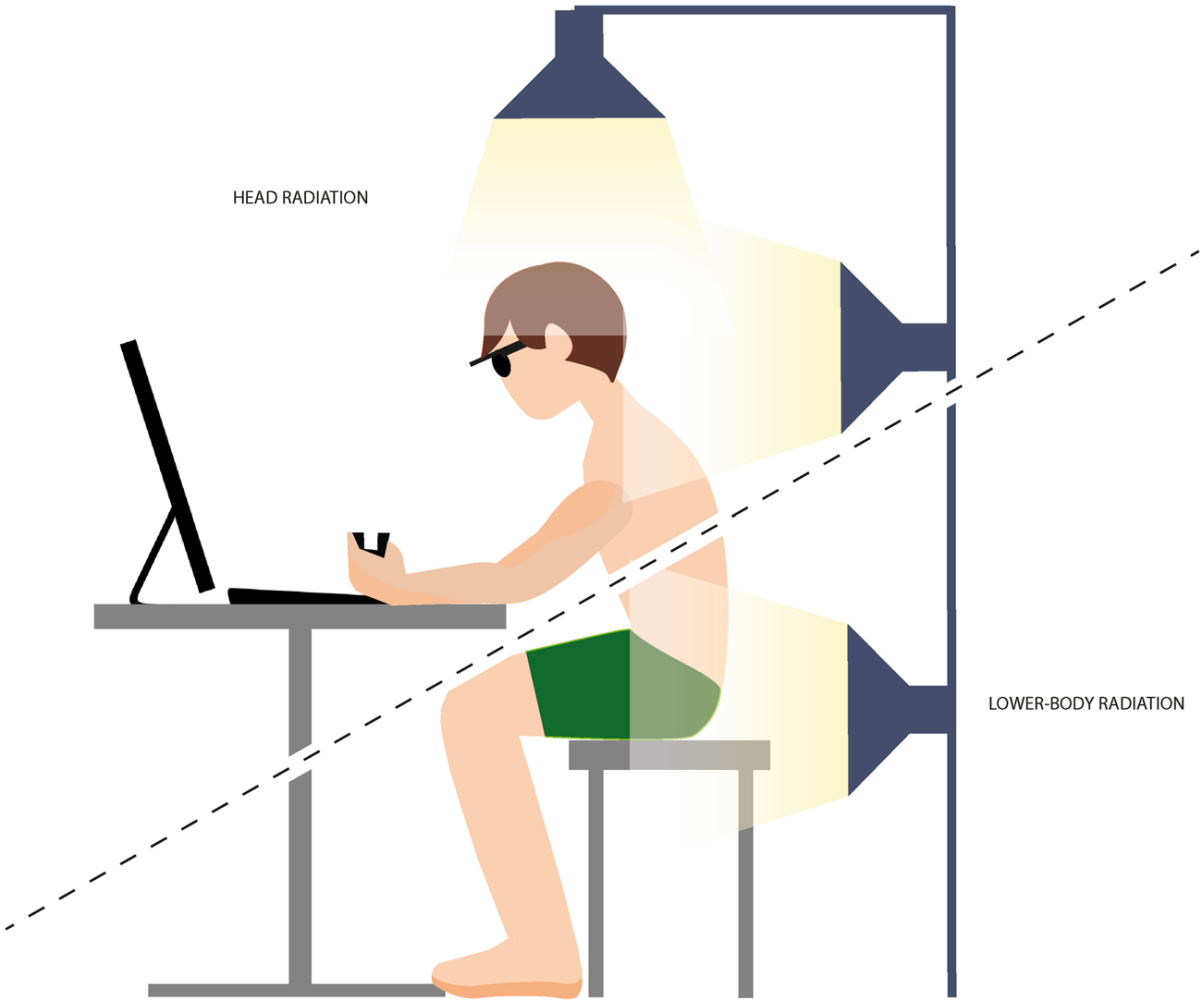
Schematic drawing of radiation setup. Four lamps was positioned (all with a distance of 50 cm away from the lower-body/head) either on the lower-body (lower right – all four lamps in the transverse plane: one on each side and two on the lower-back of the participants) or head (upper left – three in the transverse and one on top [sagittal plane]: one lamp on each side, one in the back and one on top of the participants head and neck. The dashed line symbolized the two session.

2 ×1500 stimuli of BAEPs was recorded, at 3 time-points (no radiation, acute and prolonged exposure) during both session (lower-body and head exposure, see Fig. 4). Silver electrodes were placed on the forehead (ground), vertex (CZ) and right earlobe ipsilateral to the click noise stimuli. The click noise was amplified (ARGON HA1, HI-FI klubben, Lystrup, Denmark) and entered through in-ear headphones (RHA s500u, RHA technologies, Glasgow G3 8TX, United Kingdom). The electrode signals were then amplified by 50.000 (P511 AC amplifier, Grass instrument, Astro-med inc. West Warwick, Rhode Island, USA), low to high pass frequency filtered 10Hz-3kHz and analog to digital converted (CED1401plus, Cambridge Electronic Design Limited, Cambridge, England) and sampled at 50 kHz. Thereafter, all recording were filtered (IIR bandpass 1^st^ order 200 to 2000) and a waveform average over the 1500 stimuli was created (Signal 6.05a x64, Cambridge Electronic Design Limited, Cambridge, England). Hereafter, all waveform averages of the BAEPs were visually inspected and peak I (PI) and peak V (PV) manually identified. Cursors were place where peaks was identified and interpeak latencies (IPLs) were calculated between PI and PV for each participants at all time-points across both sessions.

#### Properties of solar radiation setup

the solar radiation setup (see Fig. 4, for schematic drawing of the setup) consisted of four lamps (Halogen lamps, R7s, Cixi Zhongfa lamps CO., Ltd.,) each installed with a 400 W halogen bulb (Haloline R7s, 400 W, OSRAM). The halogen bulb photometrical properties was 9000 lumen, 3000 kelvin with a Ra index of 100 and with a spectral power distribution from 300–800 nm (sigmoid function towards 800 nm). Lamps illuminated 690 W/m^2^ in the spectral range 1000–1700 nm (LS122IR, Shenzhen Linshang, Technology Co., Ltd. Shenzhen City, China) and 505 W/m^2^ when measured in 400–1100 nm light spectrum (TES 1333 Solar power meter, TES Electrical Electronic Corp., Taipei, Taiwan, R.O.C.). Black Globe temperature (T_g,_) measured with 150 mm black painted copper globe, air temperature (T_a_) and relative humidity (RH %) measured in the center of the sphere created by the lamps (see Fig. 4) was 94 °C, 64 °C and ranged from 5–10%, respectively. All measurements were taken at a distance of 50 cm from the center of the sphere.

#### Rectal core temperature (T_re,_), mean skin temperature (MST) and exposed skin sites (ESS)

was measured when participants initially interring the environmental chamber (no radiation), when radiation was applied (acute exposure) and at the end of each sessions (prolonged exposure). T_re_ was measured with a rectal thermometer (Ellab Copenhagen, CTD85) inserted ~10 cm beyond the anal sphincter, ESS (i.e. HST [at the ocitipal bone] and LBST [at the lumbar spine] was measured on the skin surface using a skin temperature probe (Ellab Copenhagen, CTD85) and MST was measured on the skin surface at 4 different sites e.g. biceps, chest, thigh and calf, using iBOTTON sensors (type DS1921 H, maxium/Dallas semiconductor Corp., USA). To calculate MST a weighted average was calculated [0.3 (chest + biceps) + 0.2 (thigh + calf)]^55^.

#### Hydration

As has been done in our previous work^6,23,53^, hydration was assessed by measuring urine specific gravity (USG) before and after completing each session, using a refractometer (ANTAGO, pocket refractometer, s/no P811580, Tokyo, Japan). USG was measured to ensure that all participants were hydrated (USG ≤ 1.020 g·ml^−1^) before starting the trials and in combination with body mass assessment to track changes in hydration over time, in the respective sessions. During sessions, and to avoid (minimize) any dehydration and/or interference with the T_re_, the participants were required to drink water equal to expected sweat loss as estimated from their familiarization trials. Body mass (BM) was assessed using a platform scale (InBody 270, InBody CO Ltd).

#### Thermal discomfort comfort and sensation

were assessed using visual analog scale (VAS) ranging from 0–200 mm (low to maximum, respectively) and completed at no radiation (baseline), at acute- and after prolonged exposure of heat radiation and was subsequently converted to percentage from 0% (low) to 100% (maximum).

### Statistical analysis

Changes in test performance scores, BAEP interpeak latency time (peak I and peak V) and measurements across time and conditions were compared for simple motor, simple cognitive, combined motor-cognitive and complex motor tasks performances, interpeak latencies (PV-PI) and T_re_, BM, USG, HR, TDC and TS, were evaluated by a two-way repeated-measures analysis of variance test (2-way RM ANOVA) with the repeated factors of condition (3 levels: no radiation, acute exposure and prolonged exposure) and time (2 levels: lower-body and head). When a significant interaction or main effect in the 2-way RM ANOVA was found, post-hoc pairwise comparisons were performed with the Tukey correction test. All statistical analyses were carried out in GraphPad Prism (version 7.02, GraphPad Software, La Jolla, CA). Data are presented as mean ± SD (standard deviation), unless otherwise stated and the significance level was set at *P* = *0.05*.

## Supplementary information

Supplementary information.

## Data Availability

All relevant data are within the paper and its supporting information files (see excel data file).
